# Zinc finger transcription factor CASZ1 interacts with histones, DNA repair proteins and recruits NuRD complex to regulate gene transcription

**DOI:** 10.18632/oncotarget.4733

**Published:** 2015-08-07

**Authors:** Zhihui Liu, Norris Lam, Carol J. Thiele

**Affiliations:** ^1^ Pediatric Oncology Branch, National Cancer Institute, Bethesda, MD 20892, USA

**Keywords:** CASZ1, tumor suppressor, NuRD complex, DNA repair protein, cofactors

## Abstract

The zinc finger transcription factor CASZ1 has been found to control neural fate-determination in flies, regulate murine and frog cardiac development, control murine retinal cell progenitor expansion and function as a tumor suppressor gene in humans. However, the molecular mechanism by which *CASZ1* regulates gene transcription to exert these diverse biological functions has not been described. Here we identify co-factors that are recruited by CASZ1b to regulate gene transcription using co-immunoprecipitation (co-IP) and mass spectrometry assays. We find that CASZ1b binds to the nucleosome remodeling and histone deacetylase (NuRD) complex, histones and DNA repair proteins. Mutagenesis of the CASZ1b protein assay demonstrates that the N-terminus of CASZ1b is required for NuRD binding, and a poly(ADP-ribose) binding motif in the CASZ1b protein is required for histone H3 and DNA repair proteins binding. The N-terminus of CASZ1b fused to an artificial DNA-binding domain (GAL4DBD) causes a significant repression of transcription (5xUAS-luciferase assay), which could be blocked by treatment with an HDAC inhibitor. Realtime PCR results show that the transcriptional activity of CASZ1b mutants that abrogate NuRD or histone H3/DNA binding is significantly decreased. This indicates a model in which CASZ1b binds to chromatin and recruits NuRD complexes to orchestrate epigenetic-mediated transcriptional programs.

## INTRODUCTION

Although the drosophila zinc finger transcription factor castor (human homolog, CASZ1) was originally described as a neural fate determination gene [1–4], recent studies have identified a critical role for mammalian CASZ1 during the development of a variety of different cell and tissue types. In *Xenopus*, CASZ1 is required for vascular assembly [5], and in both *Xenopus* and mice CASZ1 is required for heart development [6, 7]. In mice, late born retinal neural progenitor fate is regulated by CASZ1 [8]. In humans, *CASZ1* localizes to chromosome 1p36, a region in which loss of heterozygosity has been implicated in many types of cancers [9]. We have characterized two human isoforms of CASZ1 gene, CASZ1a and CASZ1b [10, 11]. CASZ1a, the full length isoform, comprises 1759 amino acids (AA) with 11 TFIIIA class C2H2 zinc finger; CASZ1b comprises 1166 AA that are identical to the first 1166 AA of CASZ1a but lacks the last 6 zinc fingers. CASZ1b is the more evolutionarily conserved isoform [10–12]. Loss of CASZ1a or CASZ1b expression is found in neuroblastoma tumors of patients who have a poor prognosis and functional studies indicate that restoration of either CASZ1a or CASZ1b suppresses neuroblastoma tumor growth [11–13]. Despite the important role CASZ1 plays during normal development and its loss of function contributes to tumorigenesis, the mechanisms by which CASZ1 regulates gene transcription to exert its biological functions are poorly characterized.

Transcription factors regulate gene transcription through protein-protein interactions. For example, Myc interaction with Max is required for binding to E-boxes and activation of target gene transcription; p53 recruits co-activator p300 complex to activate gene transcription; REST recruits co-repressor co-REST to repress gene transcription; IKAROS recruits NuRD to activate or repress gene transcription [14–20]. Previous studies have shown in drosophila that, Hunchback, Kruppel, Pdm1, Castor (human homolog, CASZ1) and Grainyhead participate in a transcription factor cascade in which a feed-forward stimulation and feed-back inhibition pathway ensures the precise spatiotemporal development of neuroblast progenitors in drosophila [21–23]. While castor has been shown to directly bind the promoter region of Pdm1 [21] to suppress its expression and Casz1 directly binds to the EGFL7 gene locus to stimulate its transcription [5], the co-factors recruited by CASZ1 to regulate target gene transcription remain unclear.

In this study we identify that the more evolutionarily conserved CASZ1b isoform binds to histones and interacts with NuRD complex. Interestingly, CASZ1b also interacts with DNA repair proteins. Mutagenesis of CASZ1b construct defines the NuRD and histone H3 /DNA repair protein binding regions, and disruption of either H3/DNA repair protein or NuRD binding to CASZ1b significantly decreased its transcriptional activity.

## RESULTS

### CASZ1b interacts with NuRD complex, histones and DNA repair proteins

**Figure 1:**
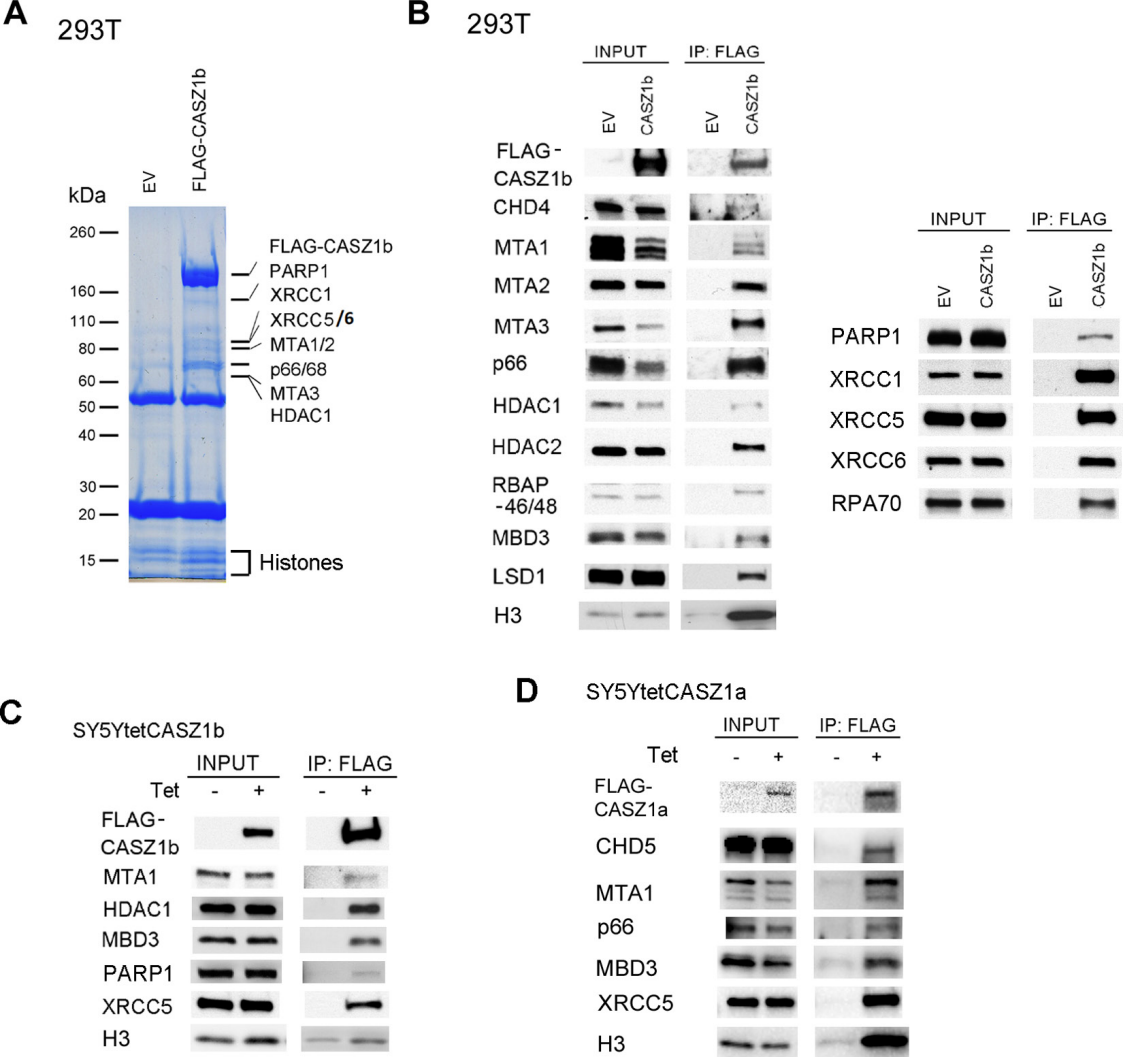
Identification of CASZ1b protein partners **A.** Empty vector (EV) or 3xFLAG-CASZ1b construct was transiently transfected into HEK293T cells, and anti-FLAG antibody was used for co-immunoprecipitation (co-IP) of the CASZ1b complex from the whole cell extracts. EV transfected cell extracts were used as control. The co-IP products were resolved by SDS-PAGE and stained with simply blue safe stain reagent. The lanes were sectioned and digested with trypsin. Extracted peptides were sequenced by mass spectrometry. **B.** EV and 1xFLAG-CASZ1b construct was transiently transfected into HEK293T cells. The cell extracts were used for co-IP with anti-FLAG antibody, and probed for subunit of NuRD and histone H3 by western blot (left panel), or probed for DNA repair proteins by western blot (right panel). **C.** Tetracycline inducible FLAG-CASZ1b construct was stably transfected into SY5Y cells, then the cells were cultured with or without 1 μg/μl tetracycline for 24 hr. The cell extracts were used for co-IP with anti-FLAG antibody, and probed for indicated proteins by western blot. **D.** Tetracycline inducible FLAG-CASZ1b construct was stably transfected into SY5Y cells, then the cells were cultured with or without 1 μg/μl tetracycline for 24 hr. The cell extracts were used for co-IP with anti-FLAG antibody, and probed for indicated proteins by western blot.

To investigate CASZ1 associated proteins, we focused on the evolutionarily conserved CASZ1b isoform. Co-immunoprecipitation (co-IP) experiments were performed on protein lysates from human embryonic kidney (HEK293T) cells that have been transiently transfected with pCMV-3Tag-3A (empty vector, EV) or 3xFLAG-CASZ1b using anti-FLAG antibody followed by separation of proteins on a 4–12% SDS-PAGE gel. After staining with SimplyBlue Safe Stain reagents, the differentially pulled down bands were sequenced using mass spectrometry. We found that proteins pulled down by CASZ1 belong to three major groups: 1. NuRD complex which is important for chromatin remodeling and gene transcription regulation; 2. histone which is a core component of nucleosome structure; 3. DNA repair proteins which are involved in DNA replication and repair (Figure 1A). To confirm the mass spec results, we performed co-IP using HEK293T cells transfected with 1xFLAG-CASZ1b and western blot analysis. We found that CASZ1b not only binds to MTA1/2/3, Gatad2a/2b (p66) and HDAC1, but also binds to other known NuRD subunits including CHD4, HDAC2 and MBD3 (Figure 1B, left panel). Mass spectrometry results showed that CASZ1b interacts with many types of histones such as HIST1H4A, HIST1H2AA and HIST1H2BB. The interaction with histone was confirmed by probing with anti-histone H3 antibody (Figure 1B, left panel). The interaction with DNA repair proteins including PARP1, XRCC1, XRCC5, XRCC6 and RPA1 was also confirmed by western blot analyses (Figure 1B, right panel). To investigate whether this interaction occurs in other cell types, we performed co-IP and western blot using the neuroblastoma SY5Y cell line (SY5Y-FLAG-CASZ1b) that is stably transfected with a tetracycline inducible FLAG-CASZ1b vector. We also found that the CASZ1b interacts with subunits of NuRD complex, histone H3 and DNA repair proteins in NB cells (Figure 1C). To investigate whether the full length CASZ1a also interacts with subunits of NuRD complex and DNA repair proteins, we performed co-IP and western blot using SY5Y-FLAG-CASZ1a and found that the pull down of CASZ1a also pulled down NuRD subunits such as MTA1 and DNA repair protein such as XRCC5 (Figure 1D). Here we also evaluated whether CASZ1a interacts with another NuRD subunit CHD5, which is a neuroblastoma tumor suppressor gene [24, 25]. We found that the pull down of CASZ1a also pulled down CHD5 (Figure 1D), indicating that in neuroblastoma cells, these two chromosome 1p36 tumor suppressors interact with each other.

**Figure 2:**
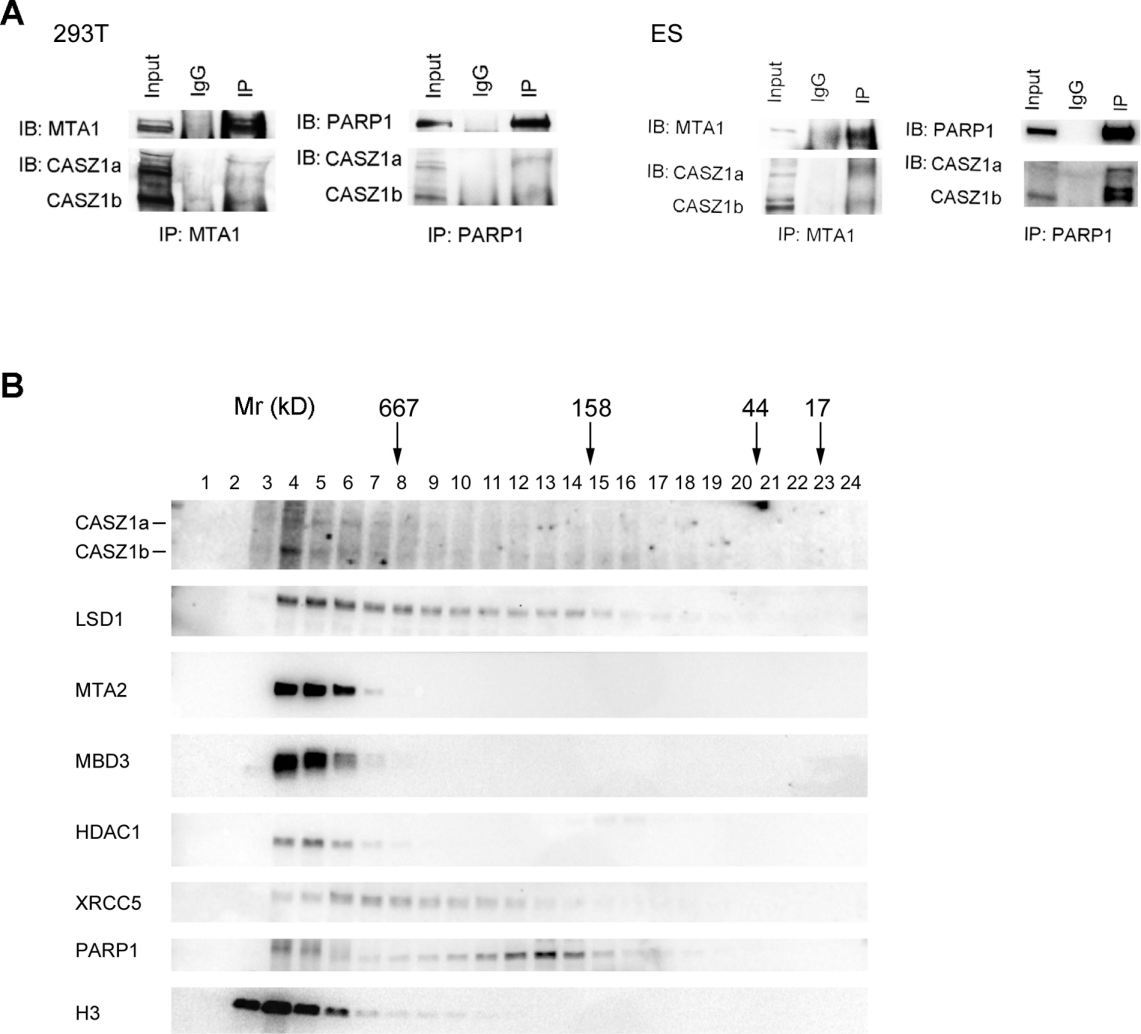
CASZ1 is associated with its protein partners *in vivo* **A.** The HEK293T whole cell extracts were used for co-IP with MTA1 or PARP1 anti antibody, and probed for endogenous CASZ1 by western blot (left panel); The mouse ES cell extracts were used for co-IP with MTA1 or PARP1 anti antibody, and probed for endogenous CASZ1 by western blot (right panel). **B.** Mouse ES cell extracts were fractionated through a gel filtration column, and selected fractions were then resolved by SDS-PAGE and western blotting performed to indentify the indicated proteins.

To investigate whether the CASZ1-NuRD complex protein-protein interaction occurred under physiological conditions, we performed co-IP using anti-MTA1 antibody or anti-PARP1 antibody in HEK293T cells. When the blot was probed using anti-CASZ1 antibody, we found that both MTA1 and PARP1 can pull down endogenous CASZ1 in HEK293T cells (Figure 2A, left panel). An *in vivo* interaction between CASZ1 and MTA1 and PARP1 was also demonstrated in mouse embryonic stem (mES) cells (Figure 2A, right panel).

To determine the composition of the CASZ1b molecular complexes, cell lysates from mES cells were subjected to size exclusion columns as detailed in Materials and Methods. The results showed that both CASZ1a and CASZ1b isoforms co-eluted with representative subunits of NuRD complex in the early fractions (>667 kD) (Figure 2B). Representative DNA repair protein XRCC5 and PARP1 also co-eluted with CASZ1 in these early fractions, but XRCC5 and PARP1 also eluted in later fractions that did not contain high levels of CASZ1 (Figure 2B). Similar to NuRD subunits, H3 predominantly co-eluted with CASZ1 in the early fractions (Figure 2B). These findings suggest that *in vivo* CASZ1 is either part of a large macromolecular complex with NuRD, DNA repair proteins and histone proteins or that CASZ1 interacts with these proteins in discrete, separate complexes with similar molecular size. Free CASZ1 (CASZ1a is ∼190 kD and CASZ1b is ∼125 kD) in the fractions that eluted in the 100–200 kD MW range is barely detected which indicates that the majority of endogenous CASZ1 forms protein complex of >667 kD in embryonic stem cells.

### N-terminus of CASZ1b is required for NuRD binding and zinc fingers are required for DNA repair protein binding

**Figure 3:**
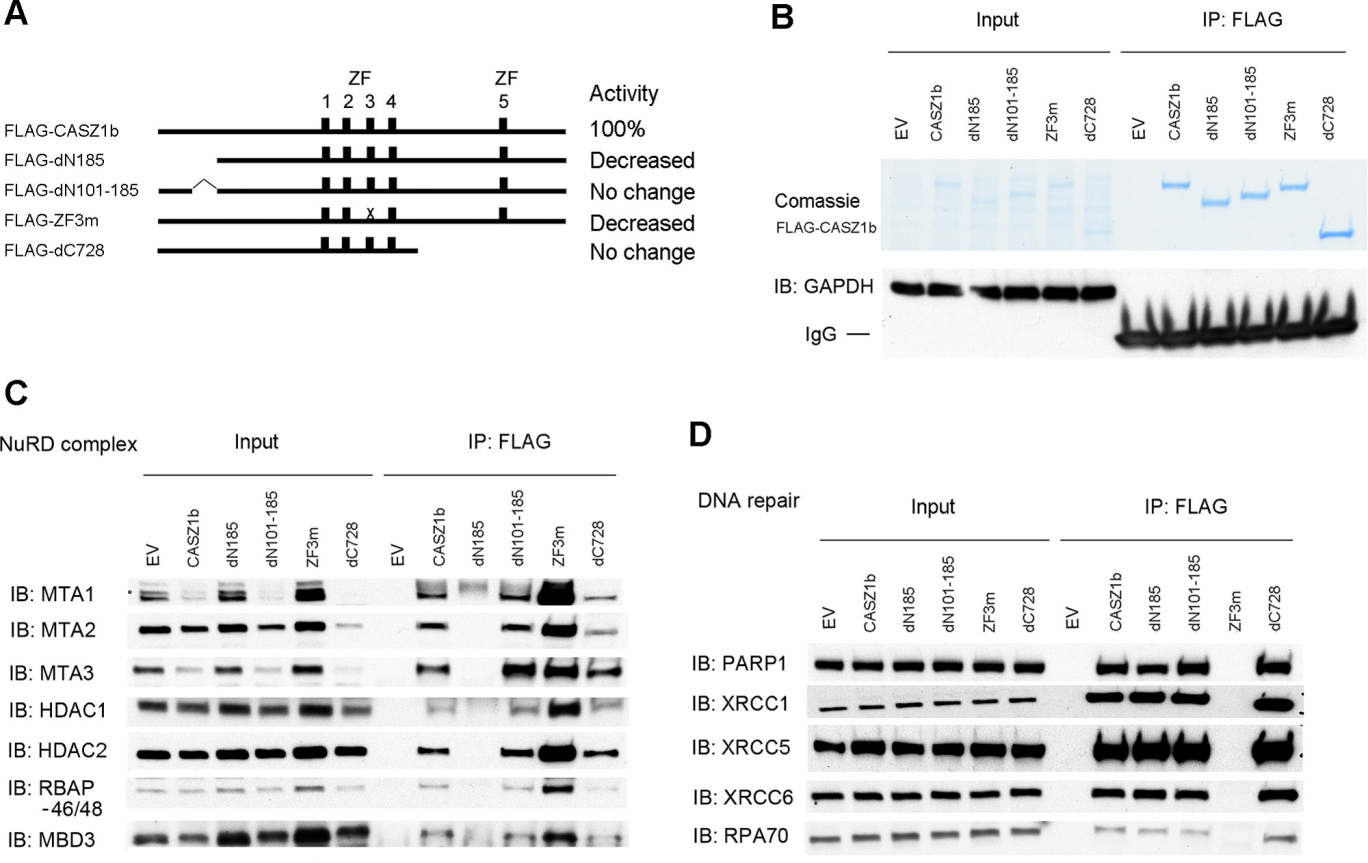
N-terminus of CASZ1b interacts with NuRD subunits and zinc finger 3 is required for DNA repair proteins binding **A.** Cartoon of CASZ1b mutant constructs (ZF represents zinc finger), to the right of the cartoon, the relative transcriptional activity of CASZ1b mutants compared to wild type CASZ1b is shown. **B.** EV, FLAG-CASZ1b, or FLAG-CASZ1b mutant construct was transiently transfected into HEK293T cells, and anti-FLAG antibody was used for co-immunoprecipitation (co-IP) of the CASZ1b complex from the whole cell extracts. The co-IP products were resolved by SDS-PAGE and stained with comassie stain to show the pull down of CASZ1b and the mutant constructs, or probed for GAPDH by western blotting to show the specificity of co-IP. **C.** EV, FLAG-CASZ1b or the mutant construct was transiently transfected into HEK293T cells. The cell extracts were used for co-IP with anti-FLAG antibody, and probed for the subunits of NuRD by western blot. **D.** EV, FLAG-CASZ1b or the mutant construct was transiently transfected into HEK293T cells. The cell extracts were used for co-IP with anti-FLAG antibody, and probed for DNA repair proteins by western blot.

Previously we had shown the N-terminus and zinc fingers 1–4 (ZF1-4) were critical for CASZ1b transcriptional activity while mutants in the C-terminus did not significantly affect CASZ1b transcriptional activity [12]. To determine which region of CASZ1b is required for protein complex binding, we evaluated the ability of CASZ1b mutants in the N-terminus, zinc finger 3 (ZF3) and the C-terminus to interact with NuRD subunits or DNA repair proteins (see Figure 3A). Co-IP using anti-FLAG antibody was able to pull down different FLAG-CASZ1b mutants but not GAPDH (Figure 3B). We found that CASZ1b N-terminus deletion mutant without amino acids (AA) 1-185 lost the ability to bind NuRD complex but retained their interaction with the DNA repair proteins (Figure 3C, 3D). Furthermore deletion of AA101-185 has no effect on the ability of CASZ1b to pull down NuRD subunits (Figure 3C), suggesting NuRD binding localizes within AA1-100. In contrast CASZ1b ZF3 mutant (cystine was replaced by alanine) lost the ability to pull down DNA repair proteins, while the binding of NuRD complex was retained (Figure 3C, 3D). A C-terminal deletion of CASZ1b, which deletes one third of the CASZ1b protein has no effect on either of these protein-protein interactions (Figure 3C, 3D).

**Figure 4:**
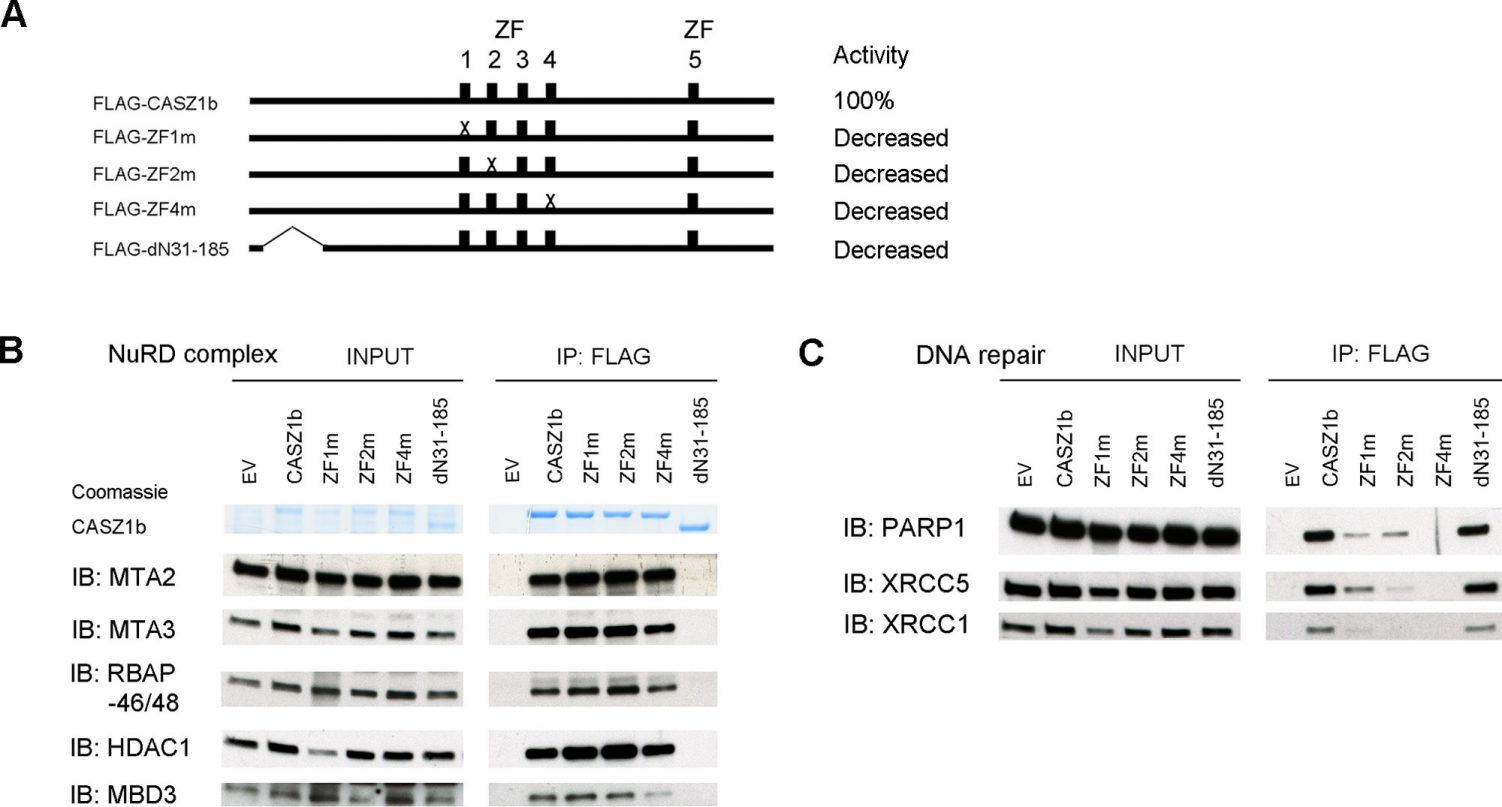
Zinc finger 1, 2 and 4 of CASZ1b is required for DNA repair proteins binding but not for NuRD subunits binding **A.** Cartoon of CASZ1b mutant constructs (ZF represents zinc finger), to the right of the cartoon, the relative transcriptional activity of CASZ1b mutants compared to wild type CASZ1b is shown. **B.** EV, FLAG-CASZ1b or the mutant construct was transiently transfected into HEK293T cells. The cell extracts were used for co-IP with anti-FLAG antibody, and probed for the subunits of NuRD by western blot. **C.** EV, FLAG-CASZ1b or the mutant construct was transiently transfected into HEK293T cells. The cell extracts were used for co-IP with anti-FLAG antibody, and probed for DNA repair proteins by western blot.

To more precisely define the regions of CASZ1b interacting with these proteins, we generated additional FLAG-CASZ1b mutants (Figure 4A). CASZ1b with N-terminal deletion of AA31-185 loses its ability to bind to NuRD complex but retains DNA repair protein binding (Figure 4B, 4C). Our ability to more precisely define this interaction region is limited because this N-terminus region is close to a CASZ1b nuclear localization site [12]. A mutation in which the cysteine of the ZF4 was replaced with an alanine completely disrupted the interaction of CASZ1b and DNA repair proteins while a similar mutation in ZF1 or ZF2 only impaired the ability of CASZ1b to pull down DNA repair proteins (Figure 4B, 4C). Unlike N-terminal deletions, zinc finger mutations did not affect NuRD complex binding to CASZ1b (Figure 4B, 4C).

### PAR binding motif of CASZ1b is required for DNA repair protein and histone H3 binding

**Figure 5:**
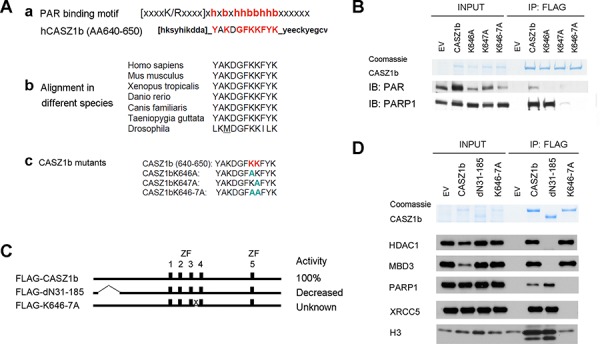
Putative poly-ADP-ribose (PAR) binding motif of CASZ1b is required for DNA repair proteins and histone H3 binding but not for NuRD subunits binding **A.** a. Putative PAR binding motif existed in the middle of CASZ1b; b. this motif is conserved in different species; c. generate CASZ1b PAR binding motif mutant constructs. **B.** EV, FLAG-CASZ1b or the PAR binding mutant construct was transiently transfected into HEK293T cells. The cell extracts were used for co-IP with anti-FLAG antibody, and probed for PAR and PARP1 by western blot. **C.** Cartoon of CASZ1b N-terminus and PAR binding mutant constructs (ZF represents zinc finger), to the right of the cartoon, the transcriptional activity of CASZ1b mutants compared to wild type CASZ1b is shown. **D.** EV, FLAG-CASZ1b N-terminus mutant or PAR binding mutant construct was transiently transfected into HEK293T cells. The cell extracts were used for co-IP with anti-FLAG antibody, and probed for NuRD subunits, DNA repair proteins and histone H3 by western blot.

Zinc fingers are important for maintaining the three dimensional structure of a protein. Disruptions of zinc fingers may change the tertiary structure of the protein and lead to the disruption of the protein-protein interaction therefore this does not necessarily mean those zinc fingers are bona-fide protein interaction or binding sites. CASZ1b contains a putative poly(ADP-ribose) (PAR) binding site (AA640-650: YAKDGFKKFYK) [26] in an evolutionarily conserved region between ZF3 and ZF4 (Figure 5A, a and b). To determine the role of PAR binding motif, we made CASZ1b PAR binding mutants by replacing the critical hydrophilic amino acids with alanine (Figure 5A, 5C). Co-IP and western blot analysis using anti-PAR or PARP1 antibody showed that CASZ1b could pull down PARylated protein, and the mutation of PAR binding motif of CASZ1b ablated this pull down ability (Figure 5B). We next transfected HEK293T cells with a CASZ1b N-terminus deletion construct (CASZ1bdN31-185) or PAR binding mutation construct (CASZ1bK646AK647A) (see Figure 5C), and performed co-IP and western blot analysis. Consistent with the previous results, the N-terminal region is required for NuRD complex binding and the PAR binding motif is required for DNA repair proteins to bind (Figure 5D). Moreover, mutation of the PAR binding motif of CASZ1b but not the N-terminal deletion disrupted interaction of CASZ1b and H3 (Figure 5D). This indicates that the PAR-binding motif of CASZ1b is required for H3 to bind.

### PARylation is not required for histone H3 to bind to CASZ1b

**Figure 6:**
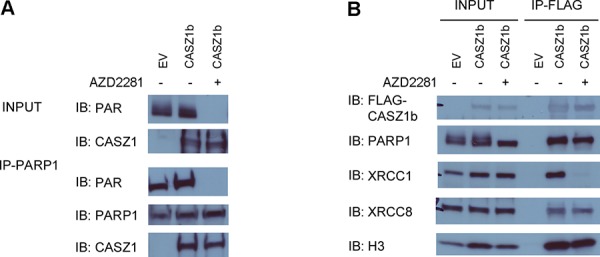
PARylation of CASZ1b is not required for histone H3 binding **A.** EV or FLAG-CASZ1b was transiently transfected into HEK293T cells with or without PARP inhibitor (AZD2281) treatment. The cell extracts were used for co-IP with anti-PARP1 antibody, and probed for PAR, PARP1 and CASZ1b by western blot. **B.** EV or FLAG-CASZ1b was transiently transfected into HEK293T cells with or without PARP inhibitor (AZD2281) treatment. The cell extracts were used for co-IP with anti-FLAG antibody, and probed for indicated proteins by western blot.

To investigate whether the interaction between CASZ1b and DNA repair protein or H3 is PARylation dependent, we transfected HEK293T cells with EV or FLAG-CASZ1b for 5.5 hr, then incubated cells in the absence or presence of the PARP inhibitor (AZD2281, 50 μM) for 24 hr. Subsequently, the cells were lysed and co-IP was performed. PARylation was completely blocked by PARP inhibitor treatment when the input blot was probed with anti-PAR antibody (Figure 6A). IP of PARP1 using anti-PARP1 antibody pulled down FLAG-CASZ1b with or without PARP inhibitor treatment (Figure 5A). Even with PARP inhibitor treatment, IP of FLAG-CASZ1b still pulled down H3 and the tested DNA repair proteins with the exception of XRCC1 (Figure 5B). This suggests that PARylation of XRCC1 is required for this protein-protein interaction. However, PARylation of other DNA repair proteins and H3 is not required for CASZ1b interaction.

### NuRD and histone H3/DNA repair proteins binding region is required for CASZ1b transcriptional activity

**Figure 7:**
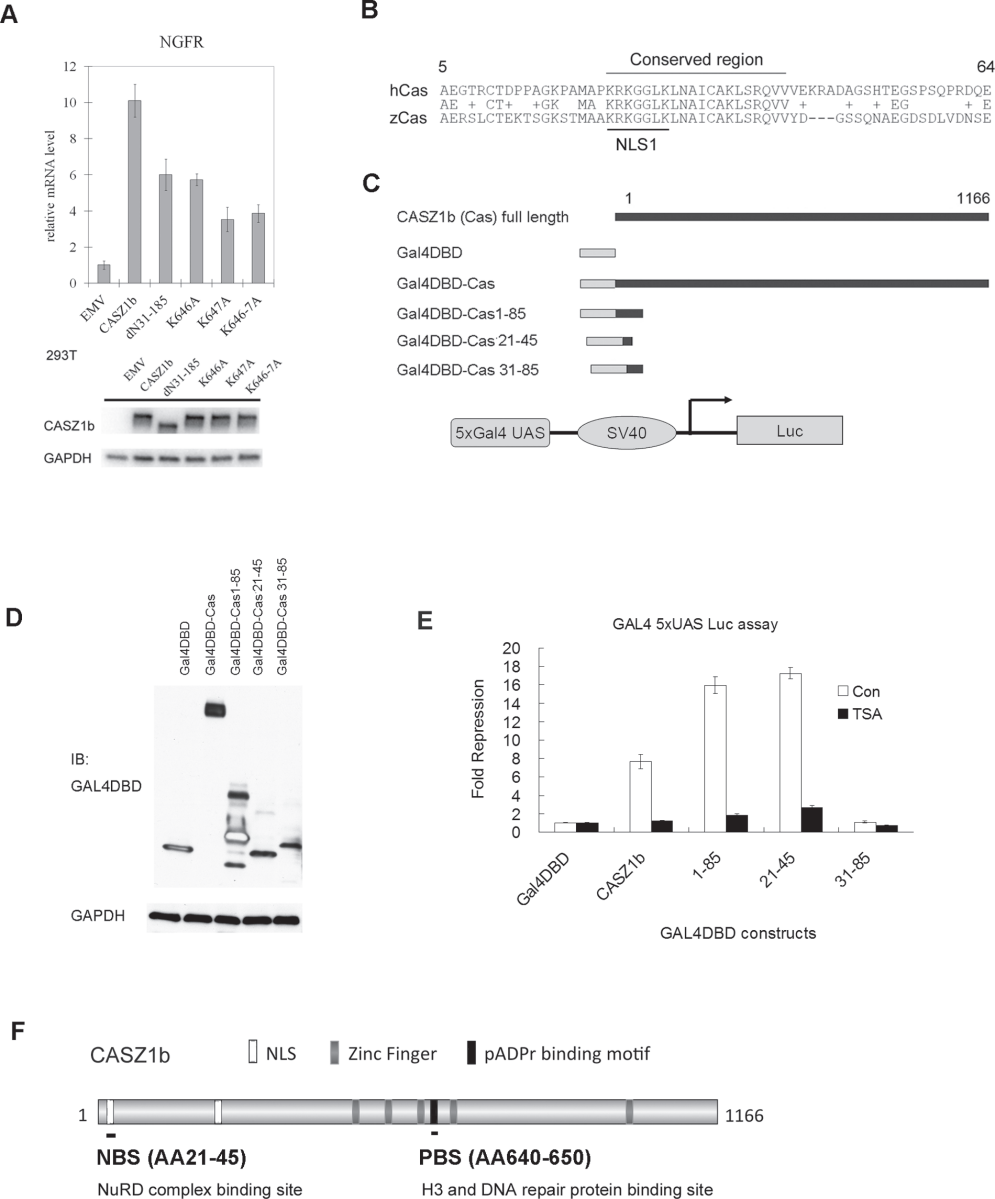
The NuRD and histone H3/DNA repair proteins binding region is required for CASZ1b transcription activity **A.** EV, FLAG-CASZ1b N-terminus mutant or FLAG-CASZ1b PAR binding mutant constructs were transiently transfected into HEK293T cells, 24 hr the cells were harvested, and the mRNA level of CASZ1b target gene NGFR was evaluated by realtime PCR (data are represented as mean ± SD). The input levels of CASZ1b and mutants were shown by western blot. **B.** The alignment of the N-terminus of human CASZ1 (hCas) and zebra fish CASZ1 (zCas). **C.** Cartoon of Gal4DBD and CASZ1b fusion protein constructs. **D.** Gal4DBD-CASZ1b fusion constructs, 5xGal4 UAS luciferase reporter vector and beta-galactosidase reporter vector) were transiently transfected into HEK293T cells, and the expression of Gal4DBD-CASZ1b fusion protein was assessed by western blot. **E.** Gal4DBD-CASZ1b fusion constructs, 5xGal4 UAS luciferase reporter vector and beta-galactosidase reporter vector were transiently transfected into HEK293T cells for 24 hr, then treated with or without HDAC inhibitor (TSA) for another 26 hr, and the cells were harvested, and the luciferase activity was evaluated using luciferase assay reagent (data are represented as mean ± SD). **F.** Schematic map of CASZ1b protein to show NuRD, H3/DNA repair proteins binding sites.

To investigate whether these protein partners are required for CASZ1b transcriptional activity, EV, CASZ1b, CASZ1bdN31-185 (NuRD binding mutant), CASZ1bK646A, K647A and K646-7A (PAR binding motif mutants, see Figure 5A) were transiently transfected into HEK293T cells for 24 hr, and realtime PCR was performed to evaluate expression of a known CASZ1b target gene - nerve growth factor receptor (NGFR). Transfection of CASZ1b WT induces a 10-fold increase in NGFR transcriptional activity but there is a significant decrease in induced NGFR transcriptional activity when either the NuRD binding mutant CASZ1bdN31-185 (6-fold increase) or the PAR binding motif mutants (4 to 6-fold increase) (Figure 7A) are transfected into HEK293T cells. The transcriptional activity of NuRD binding mutant and H3/DNA repair proteins binding mutant construct is significantly lower than wild type CASZ1b (all *p* < 0.01). This indicates that CASZ1b recruitment of NuRD complex and the ability to bind histone H3 or DNA repair proteins is required for optimal transcriptional activity.

To evaluate how the NuRD binding site of CASZ1b contributes to its transcriptional activity, we utilized the classic GAL4 DNA binding domain (GAL4DBD) and 5xUAS-reporter system [27]. The AA1-100 is required for CASZ1b to bind to NuRD complex (Figure 3), and a protein sequence alignment analysis of the N-terminus of CASZ1b revealed the region from AA23-42 containing the NLS1 signal is evolutionarily conserved among human and other species including zebrafish (Figure 7B, alignment of human CASZ1 [hCas] and zebrafish CASZ1 [zCas], around NLS1 region), highlighting a potentially important region. CASZ1b or N-terminal fragments at NuRD complex binding site were fused to GAL4DBD (Figure 7C). Different CASZ1b-GAL4DBD constructs, 5xGal4 UAS-luciferase reporter and internal control beta-galactasidase reporter vector were co-transfected into HEK293T cells (Figure 7D) and the reporter activity was assessed. Gal4DBD-CASZ1b significantly repressed transcription (7-fold, *p* < 0.005) while the Gal4DBD-CASZ1b1-85 caused a 16-fold (*p* < 0.005) repression of transcription (Figure 7E, white columns). Gal4DBD-CASZ1b21-45, which contains a short portion of the evolutionarily conserved region of the CASZ1b N-terminus, caused a 17-fold repression of transcription (*p* < 0.005). However, Gal4DBD-CASZ1b31-85, which contains a portion of the conserved region but lacks the NLS1, did not have any effect on transcription (Figure 7E, white columns). This indicates that the NLS1 region, AA21-30 is also required for CASZ1b mediated transcriptional repression. HDAC1 and HDAC2 are the two major subunits of NuRD complex [28], to determine whether the CASZ1b repressive activity is dependent on NuRD complex, we first transfected the cells with GAL4DBD fused CASZ1b constructs and 24 hr later treated cells with the HDAC inhibitor, TSA (200 nM) for an additional 26 hrs. We found TSA treatment significantly attenuated the transcriptional repression mediated by Gal4DBD-CASZ1b, Gal4DBD-CASZ1b1-85 or Gal4DBD-CASZ1b21-45 transfected cells (*p* < 0.005) (Figure 7E, black column). This suggests this activity is NuRD dependent. This is consistent with our finding that N-terminus of CASZ1b is required for NuRD complex binding (Figure 3). These findings indicate that CASZ1b represses gene transcription, and the AA23-43 region is a critical transcriptional regulatory site that operates via NuRD binding and HDAC activity to exert its role in transcriptional regulation.

Taken together, these findings indicate that CASZ1b interacts with histones, DNA protein and recruits NuRD complex to regulate gene transcription. The N-terminus AA21-45 of CASZ1b mediates NuRD complex binding, and the putative PAR-binding motif AA640-650 mediates H3 and DNA repair protein binding (Figure 7F). The disruption of either the NuRD complex or histone H3/DNA repair proteins binding significantly decreases CASZ1b transcription activity.

## DISCUSSION

Transcription factors recruit co-factors to regulate DNA accessibility in chromatin in order to modulate gene transcription [16, 29–31]. In this study, we identified NuRD complex as CASZ1 co-factors, and histones and DNA repair proteins are important CASZ1 interactors. The structure and function assays demonstrated that the N-terminus of CASZ1b is required for NuRD interaction and the putative PAR-binding motif between CASZ1b ZF3 and ZF4 is required for histone H3 and DNA repair proteins interaction. Importantly, the mutation of either of these regions significantly decreased CASZ1b transcriptional activity.

Our results showed that CASZ1b is found in a complex with NuRD proteins, indicating that CASZ1 regulates gene transcription through recruitment of NuRD complexes. The NuRD chromatin remodeling complex links multiple processes including histone deacetylation, histone demethylation, nucleosome mobilization, binding of methylated DNA and histones, and recruitment of other regulatory proteins [24, 28, 32–35]. NuRD complex subunits include chromodomain helicase DNA-binding (CHD) protein CHD3/CHD4/CHD5, histone deacetylases (HDAC)1/2, histone demethylase LSD1, metastasis-associated proteins (MTA)1/2/3 and methyl CpG binding domain proteins (MBD)2/3. NuRD influences cell fate decisions by modulating transcriptional activity through interacting with sequence specific DNA binding factors [32]. Although originally identified as a transcriptional repressor complex, components of the NuRD complex bind to some actively transcribed genes [18, 19, 36] and are thought to provide a mechanism for fine-tuning transcription during development. Our transcriptome analysis of normal and Casz1 knockout hearts from E12.5 mice showed that 64% of genes was repressed by CASZ1 and 36% of genes was activated by CASZ1 [7], indicating that CASZ1 may function as either a transcriptional activator or repressor depending on target genes. Although the Gal4DBD-CASZ1b and 5xGal4 UAS-luciferase reporter assays indicate that CASZ1 may act primarily as a transcriptional repressor through its recruitment of NuRD (Figure 7), it is also possible under different cellular chromatin environments that CASZ1 may recruit NuRD to activate gene transcription.

We have mapped the interaction of NuRD to sequences in the NH2 terminal portion of CASZ1, while we find that CASZ1 interacts with H3 via a PAR-binding motif in the zinc finger region. Mutation of the PAR-binding motif of CASZ1b attenuated H3 binding and significantly decreased CASZ1b's transcriptional activity of CASZ1b (Figure 7A). At this point we cannot distinguish whether all or a subset of the interactors that bind this region are required for transcriptional activity. CASZ1b binding to histone H3 suggests a requirement to localize to certain chromatin structures to modulate gene transcription.

Mutation of the PAR-binding motif also attenuated the interaction of CASZ1b to DNA repair proteins. Single or double AA mutation of the PAR binding motif in CASZ1b significantly decreased the ability of CASZ1b to interact with DNA repair proteins indicating that this motif is required for DNA repair protein binding to CASZ1 (Figure 5). The finding that ZF1-4 mutants also disrupt the interaction between CASZ1b and DNA repair proteins suggests that the natural three dimensional structure of CASZ1b in this region is also important for this interaction since zinc finger domains are generally required for maintaining the three dimensional structure. It is also possible that each of ZF1-4 of CASZ1 is required for DNA binding, and the binding to DNA is required for CASZ1 to interact with DNA repair protein. The finding that inhibition of PARylation disrupts the interaction of CASZ1 and XRCC1 (Figure 6B) suggests that PARylation of XRCC1 and perhaps other proteins may be required for its interaction with CASZ1. However PARylation is not required for the interaction between CASZ1 and some other proteins such as histone H3 (Figure 6B).

The interaction of CASZ1 with DNA repair proteins and the epigenetic modifier NuRD complex may link gene transcription and the DNA repair process. In fact, the “interactomes” of other transcription factors such as SOX2 and OCT4 also have the capability to bind NuRD complex, PARP and DNA repair proteins [37–39]. DNA repair proteins influence transcription [40, 41] and also participate in transcription coupled DNA repair, in which the lesions from template DNA strands of actively transcribed gene can be removed [42]. We have found that CASZ1 co-elutes with NuRD subunits and the DNA repair proteins PARP1 and XRCC5 (Figure 2), which suggests that these proteins may be able to form a large complex involved in transcription-coupled DNA repair or DNA repair-coupled gene transcription regulation. Consistent with this, PARP has been shown to recruit two components of the repressive NuRD complex, CHD4 and MTA1 to DNA lesions thus setting up a transient repressive chromatin structure at sites of DNA damage to block transcription and facilitate DNA repair [43]. To determine how CASZ1 and its associated proteins cooperate to regulate gene transcription, our future studies will examine the localization of CASZ1 and its associated proteins such as NuRD complex and histones on genomic DNA by ChIP-sequencing. To our knowledge, this is the first study to identify CASZ1b associated proteins that modulate its transcriptional activity. These findings are important to understand how CASZ1 regulates gene transcription and exerts its biological function. Since we only analyzed the most differentially expressed proteins, we have only a partial view of the CASZ1 interactomes (Figure 1A) and our future experiments will include a comprehensive analysis of all interactors.

We have gained insights into how CASZ1 may function as a transcription factor and tumor suppressor. Components of Mi-2/NuRD complex MTA1/2/3, MBD3 and LSD1 have been linked directly to oncogenesis [33, 34]. Both CASZ1 and NuRD subunit CHD5 have been identified as chromosome 1p36 tumor suppressor genes in neuroblastoma, and both are frequently lost in high-risk neuroblastoma patients [9, 13, 25]. Our finding that CASZ1 interacts with CHD5, a component of NuRD complexes indicate they function in a common pathway critical for regulating growth of progenitors involved in sympathoadrenal differentiation. CASZ1 also interacts with DNA repair proteins, which are required to maintain the genome stability and is essential to successfully complete cellular division and avoid tumorigenesis [44]. Thus CASZ1 may work with DNA repair proteins to stabilize genomic DNA and avoid carcinogenesis during normal development.

The endogenous interaction of CASZ1b and its associated proteins were demonstrated in 293T cells and mES cells (Figure 2), but was not investigated in NB cells since CASZ1 levels are low due to 1pLOH in this region as well as epigenetic silencing of CASZ1. Nevertheless, at steady-state, CASZ1 interacts with proteins involved in chromatin formation, chromatin remodeling, DNA replication and repair. Whether CASZ1 interacts with these proteins solely as a large complex and whether the composition of these interactions change as the chromatin environment reprograms during differentiation remain to be elucidated. However, the discovery of these interactions provides insights relevant for delineating the mechanisms underlying the function of CASZ1 during neurogenesis, heart development, vascular assembly and tumorigenesis.

## MATERIALS AND METHODS

### Cell culture

Human embryonic kidney cells (HEK293T) were obtained from ATCC. HEK293T cells were maintained in Dulbecco's modified Eagle's media supplemented with 10% fetal calf serum as well as 100 μg/mL streptomycin, 100U/mL penicillin, and L-glutamine. Neuroblastoma SY5YtetCASZ1b stable clone with tetracycline inducible CASZ1b expression [11] was maintained in RPMI1640 supplemented with 10% fetal calf serum as well as 100 μg/mL streptomycin, 100U/mL penicillin, L-glutamine, 500 μg/mL Geneticin (Invitrogen) and 5 μg/mL Blasticidin (Invitrogen). Mouse embryonic stem (mES, E14.Tg2a) cells were cultured in ES culture medium containing 10% FBS and LIF. All the cells were grown at 37°C with 5% CO_2_ and were passaged at 70–80% confluence 2–3 times per week.

### Construction of CASZ1b mutant constructs and CASZ1b-GAL4DBD fusion constructs

The pCMV-FLAG_CASZ1b plasmid was generated by cloning CASZ1b into pCMVTag2A as previously described [10]. To generate 3xFLAG-CASZ1b construct, CASZ1b cDNA was cloned into pCMV-3Tag-3A vector (Agilent). The QuikChange XL Site-Directed Mutagenesis Kit (Stratagene) was utilized to introduce various missense mutations into the wild type pCMV-FLAG_CASZ1b cDNA plasmid as per the manufacturer's protocol [12]. Also, some of the CASZ1b mutant constructs used here were produced in previously study [12]. A 3xFLAG-CASZ1b construct was used for co-IP and mass-spectrometry assay, and for all the other studies, 1xFLAG-CASZ1b construct or 1xFLAG-CASZ1b mutant constructs were used. To make the Gal4DBD and CASZ1b fusion constructs, different fragments of CASZ1b were generated by PCR amplification of the appropriate region prior to cloning. The primers used were synthesized with an attached 5′ EcoRI site and a 3′ NotI site and each fragment cloned into the corresponding site of Gal4DBD empty vector (EV) that provided by Dr. Philip Tucker [27]. The mutants were sequence-verified prior to analysis. The oligonucleotide primers used in the mutagenesis reactions will be provided upon request.

### Transient transfections and luciferase assay

For co-immunoprecipitation (co-IP), mRNA and luciferase experiments, plasmids were transiently transfected into HEK293T cells using the Lipofectamine 2000 cationic lipid reagent (Invitrogen) according to the manufacturer's protocol. The 5xGal4-UAS-luciferase (5xUAS-luc) reporter in pGL2 vector was generously provided by Dr. Philip Tucker [27]. A CMV-driven β-galactosidase construct was co-transfected with 5xUAS-luc and different Gal4DBD-CASZ1b mutants in order to provide an internal control for transfection efficiency. Luciferase activity was quantified using the Luciferase Reporter Assay System (Promega), and beta-galactosidase activity was measured concomitantly with the Luminescent Beta-galactosidase Detection Kit II (Clontech) to normalize the luciferase signals. To investigate whether the CASZ1b repression activity is HDACs dependent, Gal4DBD fused CASZ1b constructs were transiently transfected into 293T cells for 24 hr, and then the cells were treated with HDACs inhibitor Trichostatin A (TSA) for 26 hr. The luciferase experiments in triplicates have been repeated at least twice.

### RNA isolation and cDNA analysis by qRT-PCR

Total mRNA was collected 24 hours after transfection using the RNeasy Mini Kit (Qiagen) as per the manufacturer's protocol. Endogenous transcription of known neural differentiation-associated CASZ1 target genes, nerve growth factor receptor (NGFR) [13] was used to assess transcriptional activity of CASZ1b variants. Quantitative measurements of total β-actin and NGFR levels were obtained using an ABI StepOne plus Sequence Detection System thermocycler in triplicate. Ct values were standardized to β-actin levels, and the fold change in mRNA was calculated compared to the pCMV-Tag2A (empty vector, EV) transfected control samples. Student's *t*-test was used to assess statistical significance.

### Co-immunoprecipitation (co-IP), mass spectrometry assay and western blot analysis

For co-immunoprecipitation, HEK293T cells in 10 cm dishes were transiently transfected with different plasmids. After 24 h the cells were washed once with PBS, and resuspended in 5 ml cold PBS with PMSF (1 mM) and centrifuged at 400 g at 4°C. Cells were solubilized for 30 min in 0.6 ml cold lysis buffer (50 mM Tris-HCl pH 8.0, 200 mM NaCl, 1% Triton X-100, 1 mM dithiothreitol, 1 mM EDTA) supplemented with 50 U/mL benzonase (Novagen) and protease and phosphatase inhibitors (Halt protease and phosphatase inhibitor, Thermo), by shaking at 4°C. Whole cell extracts were clarified by a 10 min centrifugation at 20,000g at 4°C. The clear cell lysate was incubated for 4 hr with ANTI-FLAG M2 Magnetic Beads (Sigma-Aldrich) and agitated at 4°C. Subsequently the beads were washed 5 times with a 1 ml wash in a 50 mM Tris-HCl pH 8.0, 200 mM NaCl, 1% Triton X-100, 1 mM EDTA buffer. The co-IP products were eluted by incubating with 2x SDS loading buffer and boiling for 3 min. The eluted proteins were separated by 4–12% SDS-PAGE gel. After staining with SimplyBlue Safe Stain reagents, the differentially pulled-down bands were sequenced using mass spectrometry (NCI-Frederick protein analysis core facility). To perform co-IP experiments in SY5YtetCASZ1b cells, the same protocol as detailed above was used but without transient transfection. To detect endogenous interaction of CASZ1 and its protein partners, anti-PARP1 antibody and anti-MTA1 were incubated with Dynbeads (Invitrogen), and similar procedures were performed. For western blot analysis, anti-FLAG M2 monoclonal antibody (Sigma), anti-Gal4 antibody, anti-GAPDH antibody (Santa Cruz Biotechnology), anti-HDAC1, HDAC2, PARP1, XRCC1, XRCC5, XRCC6, RPA, RBAP46/48, CHD4, PAR, MBD3 antibody (Cell Signaling), anti-MTA1, MTA2, MTA3 antibody (Bethyl), were used as primary antibody. Protein bands were detected using a goat anti-rabbit or mouse IgG-HRP conjugated secondary antibody (200 μg/mL; Santa Cruz Biotechnology) and visualized using enhanced chemiluminescence (Amersham Biosciences).

### Size exclusion

Mouse embryonic stem cells were solubilized and extracted using the same lysis buffer and procedure as detailed above for the total protein for co-IP assay. The extracted proteins were separated on HPLC using a Sepax SRT SEC 300 column. Separated samples were run through 4–20% SDS-PAGE gel, and western blot analysis were performed to identify the fractions containing indicated proteins.

### Statistical analyses

Statistical analyses of the data were performed using a *t*-test with *P* < 0.05 considered significant. Values in the graphs are expressed as means ± SD. The statistical tests were two-sided.
